# Design of a Consumer Mobile Health App for Heart Failure: Findings From the Nurse-Led Co-Design of Care4myHeart

**DOI:** 10.2196/14633

**Published:** 2019-09-23

**Authors:** Leanna Woods, Jed Duff, Erin Roehrer, Kim Walker, Elizabeth Cummings

**Affiliations:** 1 School of Nursing University of Tasmania Darlinghurst Australia; 2 St Vincent's Private Hospital Sydney Darlinghurst Australia; 3 School of Nursing and Midwifery University of Newcastle Newcastle Australia; 4 School of Technology, Environments and Design University of Tasmania Hobart Australia; 5 School of Health Information Science University of Victoria Victoria, BC Canada

**Keywords:** heart failure, mobile health (mHealth), mobile apps, self-management, mobile phone, patient involvement

## Abstract

**Background:**

Consumer health care technology shows potential to improve outcomes for community-dwelling persons with chronic conditions, yet health app quality varies considerably. In partnership with patients and family caregivers, hospital clinicians developed Care4myHeart, a mobile health (mHealth) app for heart failure (HF) self-management.

**Objective:**

The aim of this paper was to report the outcomes of the nurse-led design process in the form of the features and functions of the developed app, Care4myHeart.

**Methods:**

Seven patients, four family caregivers, and seven multidisciplinary hospital clinicians collaborated in a design thinking process of innovation. The co-design process, involving interviews, design workshops, and prototype feedback sessions, incorporated the lived experience of stakeholders and evidence-based literature in a design that would be relevant and developed with rigor.

**Results:**

The home screen displays the priority HF self-management components with a reminder summary, general information on the condition, and a settings tab. The health management section allows patients to list health care team member’s contact details, schedule medical appointments, and store documents. The My Plan section contains nine important self-management components with a combination of information and advice pages, graphical representation of patient data, feedback, and more. The greatest strength of the co-design process to achieve the design outcomes was the involvement of local patients, family caregivers, and clinicians. Moreover, incorporating the literature, guidelines, and current practices into the design strengthened the relevance of the app to the health care context. However, the strength of context specificity is also a limitation to portability, and the final design is limited to the stakeholders involved in its development.

**Conclusions:**

We recommend health app development teams strategically incorporate relevant stakeholders and literature to design mHealth solutions that are rigorously designed from a solid evidence base and are relevant to those who will use or recommend their use.

## Introduction

The management of chronic conditions is an important public health challenge [1]. Globally, 26 million people live with heart failure (HF) [2], a chronic condition with considerable economic burden [3] that places great stress on patients, caregivers, and health care services [2]. Supporting patients and caregivers in long-term HF care is essential [2] with self-management linked to better quality of life, lower mortality, and readmission rates [4]. For these reasons, self-management is supported by health care policy [5,6] and is the mainstay for disease management in HF [4,7]. However, as with many chronic conditions, patients with HF find it difficult to follow self-care advice because it can be complex and challenging to sustain behavior change over the long term [4].

In an era of rapid technological advancement, there is a growing interest in consumer digital health to help with improving health. Out of the 318,000 plus mobile health (mHealth) applications (apps) available to consumers across the world [8], an abundance of health apps are available for self-monitoring [5] with condition management apps now accounting for 40% of apps [8]. The widespread interest among patients with chronic conditions to use health technologies stretches across health status, age, and other sociodemographic variables [9]. The quantity and variety of mHealth apps available present an overwhelming choice for consumers [8,10], often without guidance from their health care provider [10].

From the health provider perspective, the lack of evidence regarding the effectiveness of mHealth apps to improve health care outcomes limits their addition to treatment protocols [10]. Particular concerns are around the evidence of consumer apps regarding accuracy, efficacy, and security [10], and the inconsistent impact on disease control and health care utilization [11]. Most apps are developed outside health care systems [10], the average app quality is often low [8], and some may even threaten patient safety and privacy [12]. mHealth apps are neither yet established for widespread and sustained use nor embedded in the Australian health policy [5]. More locally, our health service’s HF team does not currently recommend a HF self-management app to patients. However, the body of evidence regarding the health impacts of mHealth apps is expanding [5,8], exampled by the growing number of clinical trials in recent years [10], and the value of mHealth to improve health care delivery is high among providers [10].

If we are to embrace consumer digital health care for its potential to address the burden of chronic conditions, interventions need to be well designed, evidence-based, and fit-for-purpose for health care providers and health care consumers alike. With this in mind, the aim of this research was to use co-design processes to develop a consumer mHealth intervention for HF self-management that is both relevant to stakeholders and developed with rigor. This paper reports the outcomes of the nurse-led design process in the form of the features and functions of the developed app, *Care4myHeart*.

## Methods

### Methodology

This research was informed by the Design Science Research Cycles proposed by Hevner [13] and refined by the research team [14]. Hevner’s framework consists of 3 cycles: design, relevance, and rigor. The relevance cycle consists of context-specific inputs from the environment, and the rigor cycle incorporates theories and methods from the existing knowledge base [13]. Data from both cycles were incorporated into the design cycle where the innovation was developed and iteratively refined [13].

### Design Process

The systematic design and development followed the Stanford University’s Design Thinking Process of innovation [15]. The 5-stage process enlisted incorporated empathizing with stakeholders, defining the health care challenge, ideating possible solutions, creating a rapid prototype, and testing with end users [15]. Embedded in the research is co-design. Co-design is a design-led process incorporating creative and participative principles and tools to actively involve a diverse group of stakeholders to explore, develop, and test solutions to shared challenges [16]. Clinicians, patients, and family caregivers were recruited from our health service, a large metropolitan tertiary hospital campus specializing in cardiac care in metropolitan Sydney, Australia. Clinicians included 2 nurse practitioners, 1 nurse consultant, a dietitian, a physiotherapist, a pharmacist, and a cardiologist. Design activities were led by a cardiac clinical nurse specialist and occurred on the hospital campus or via email as required. Ethical approval was granted from the University of Tasmania and St Vincent’s Private Hospital Sydney. First, we present the design processes enlisted in the empathize and define phase, followed by creative, dynamic processes within the ideate and prototype phase.

### Empathize and Define

Interviews were conducted with 7 patients, 4 family caregivers, and 7 clinicians to identify experiences, challenges, and opportunities regarding the lived experience of the main stakeholders. The following design artefacts—material objects that can be viewed by others, used to challenge perceptions, and inspire new ideas [17]—were created by the research team from analysis of the data:

1. Journey map: a list of daily self-care activities and associated emotional responses.
2. Stakeholder map: personal and professional persons involved in self-care.
3. Personas: 4 diverse characters representing patient needs and insights [18].
4. Current care summary: health professional’s critique of self-care support [19].
5. Clinical relevance information: considerations for the effective implementation of the mHealth app [19].

The design brief was developed by the research team (authors 1, 2, 4, and 5) from analyzing the design artefacts. It is a result of the composite of the design artefacts as interpreted by the research team. The design priorities within the brief were to (1) address medication and symptom management challenges, (2) involve some form of self-care plan, and (3) manage all stakeholders in care, as well as being evidence-based, useful, simple, and easy to use [19].

### Ideate and Prototype

A subset of 11 participants representing each of the three stakeholder groups (seven clinicians, three patients, one family caregiver) participated in two workshops and 4 months of iterative prototype development in 2017 [20]. This subset of participants will hereafter be referred to as *co-design team members*. Firstly, design artefacts were actively used in timed and focused group activities within the workshops resulting in a storyboard of the initial design on a whiteboard. Thereafter, individual co-design team members met with the design lead to refine the prototype referring to the design artefacts and other resources as required. A recurrent analysis of the academic literature, local policies, national guidelines, standards, online resources, and self-management tools ensured consistency with the evidence base. Co-design team members identified these resources as needed and referred to them intermittently throughout the development. The skills, knowledge, and experience of each co-design team member was incorporated in version updates which involved an ongoing and collaborative negotiation between co-design team members to decide on the content. The final software version reported in this paper represents the outcomes of the design cycle as the team’s collective decisions regarding the features and functions of the app.

## Results

The findings elicited throughout the co-design process are reported alongside each app component in a justification of the final app design. First, we present an overview of the app and thereafter describe the app’s 3 main sections.

### Design Overview

**Figure 1:**
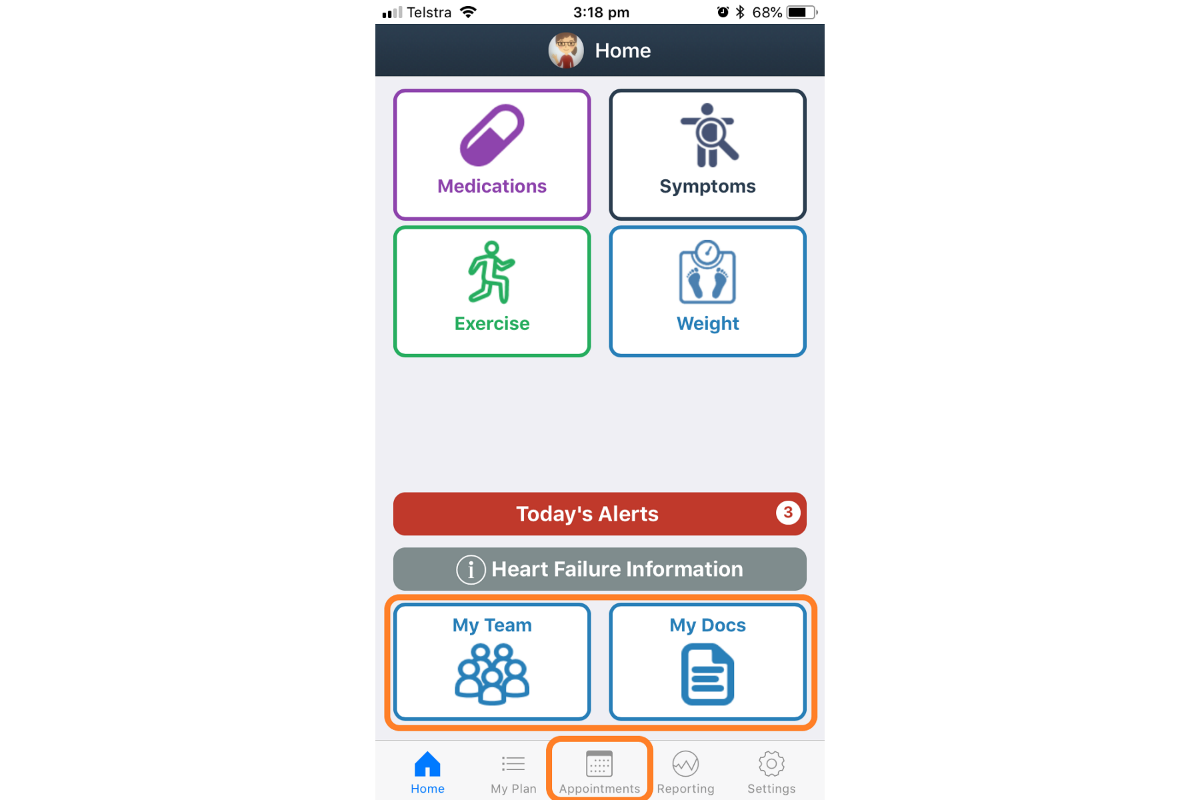
The Care4myHeart home screen including the health management section (boxed in orange).

**Figure 2:**
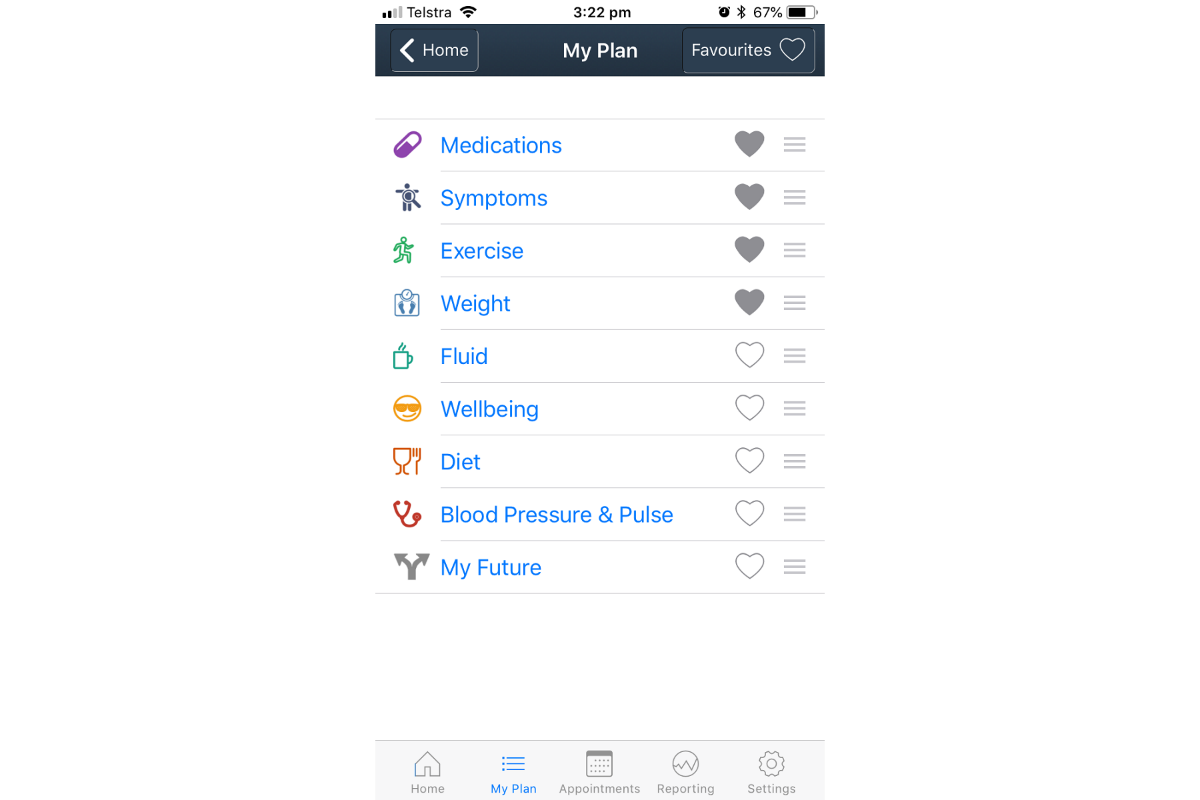
The Care4myHeart app "My Plan" section.

*Care4myHeart* is an evidence-based, modular, patient-facing consumer mHealth app for Android and iOS. The app interface consists of 3 main sections: (1) the *home screen*, (2) the *health management* section, and (3) the *My Plan* section. The home screen is the initial contact with the app’s interface and contains the priority and daily components of HF self-management, reminder summary, general information on the condition and a settings tab (see Figure 1). As much of the self-management work for patients with long-term conditions is associated with management of medical documentation, medical appointments, and health care team interactions, health management tools are included in the app’s design and are reported under the heading *health management* (see boxed sections, Figure 1). The *My Plan* section includes nine components of HF self-management and the favorites option (see Figure 2).

The three main sections of the app are described further with a description of the rationale behind the design.

### Section 1: Home Screen

Table 1 presents the home screen design which comprises the *My Plan* subsection, HF information, today’s alerts, and settings.

**Table 1:** Care4myHeart’s home screen design and rationale.

Subsection	Item	Rationale
My Plan icons	Nine self-management components; *favorites* appear on home screen	Design priority to involve some kind of self-care plan; clinicians wanted individualized care plan which involves the patient and family; standards [21] and recommendations [7] for the ongoing management of HF^a^.
Heart failure information	Information pages: overview, symptoms, and treatments	Design priority to have an evidence-based resource that would be useful, simple, and easy to use; clinicians wanted early, regular, clear, appropriate, basic, and needs-based educational material; health literacy considerations; the credible source for the information was the St Vincent’s Heart Health website [22].
Today’s alerts	List of tasks to be completed	The literature highlights the key measures to track in HF and the importance of setting self-care goals [23-25].
Settings	Enter baseline data and set goals	The team referred to the key measures to track in HF and recommendations to set self-care goals [23-25].

^a^HF: heart failure.

#### My Plan

This subsection is based on the principles within the Australian standards [21] and recommendations [7] for the ongoing management of HF which emphasize the need for self-care education and support [7]. The core requirements of these standards are the provision of clear and reliable information on symptoms, exacerbating factors, and both medical and lifestyle management [7]. The benefit of *My Plan* subsection is the modular approach providing an option as to which subsections are pertinent, in appreciation that individually tailored management plans are recommended as a tool to support care coordination [21] and optimize wellness. For relevance to the health care context, clinicians believed individualized care planning could be improved in current practice with a key design priority to involve a self-care plan. The favorites function—allowing users to select their individual priority *My Plan* components—displays important self-management subsections on the home screen. Having favorites displayed on the home screen was especially relevant as it actively facilitates the involvement of patients and family in individualized care planning during the set-up process, through choice of components from the *My Plan* list *and* number of components based on their preferences and goals.

#### Heart Failure Information

HF information, written in plain English including an overview of the condition, common symptoms, and treatments, is found via a button on the home screen accessible by patients and their family. The information was sourced from the St Vincent’s Heart Health website [22] and deemed an appropriate inclusion by clinicians and patients alike. The HF information section provides an opportunity to communicate educational material in *patient-friendly* language as a useful, clearly displayed repository of evidence-based information, as prioritized by clinicians and noted in the design brief. Clinicians recognized that educational material for patients with HF should be given early, regularly and should be clear, appropriate, basic, and needs-based. Previous work with health writers for the website content was discussed in the design workshops, and thus, paragraph sizes were limited to 4 to 5 lines, and large text sizes were used to improve readability for patients. Providing a HF information summary clearly visible on the home page was deemed important by the co-design team who often encounters family members asking for details about the condition and its treatments.

#### Today’s Alerts

A summary list of self-management tasks for the user to complete for the day (today’s alerts) was a priority design inclusion. Local clinicians believed that follow-up with patients should be improved in current care to aid memory. The co-design team members considered reminders and scheduling important functions of the app to be addressed and are, therefore, included features in the alerts summary.

#### Settings

Baseline self-management data and patient goals are personalized in the app’s settings. The co-design team members prioritized patient ownership, interactivity, and tracking as important for the user experience. In the app settings, the key measures to track [23-25] or goals to be set relate to weight, fluid restriction volume, blood pressure (BP), pulse, daily steps, and number of exercise videos to be viewed daily.

### Section 2: Health Management

The *health management* section of the app provides the opportunity for users to enter medical appointments into a calendar, digitally store medical documents, and list contact details for all personnel involved in their care. The design and rationale are summarized in Table 2.

**Table 2:** The health management section design and rationale.

Subsection	Item	Rationale
Appointments	Add medical appointments including detail	Patients experienced challenges managing multiple appointments with family caregivers often assisting; clinicians believed follow-up and connected care is not done well in the health service; scheduling and reminders were a priority; reviewed current tools for documenting clinic and doctor visits [23,25].
My Docs	Store, review, and share test results, letters, and referrals	Some patients wanted test results but may misplace documentation; clinicians wanted to include or track data, facilitate team communication, and maximize and join care; reviewed tools to document health records [23,25]; hospital’s discharge checklist contains echocardiogram results [21].
My Team	Contact details of emergency contact person and health professionals	Patients frequently liaise with their health care team but experience poor information sharing between health care providers; a design priority was to manage all stakeholders in care well and facilitate team communication; referred to the recommendations [7] and standards [21] for multidisciplinary care in HF^a^; the literature highlights the importance of team communication [24] and provides tools to document their contact details [23-25]; the hospital’s HF discharge checklist contains postdischarge care and follow-up details [21]

^a^HF: heart failure

#### Appointments

The appointments tab contains a calendar to add, review, and set reminders for medical appointments with the ability to add detail needed for the appointment. The HF self-management literature lists the importance of keeping track of clinic and doctor visits [23,25]. Locally, clinician’s critique of current self-management support is that follow-up care and connected care is not done well, and the co-design team prioritized *scheduling and reminders* as design priorities. From the patients’ perspective, there were reported challenges managing multiple medical appointments as some choose to take notes immediately after appointments to summarize the conversation to capture the complexity of care. Especially, necessary for those living in rural areas who need to travel for specialized medical care, careful coordination of appointments effectively could improve time away from loved ones, avoid early wake-ups, and missing meals or medication doses. Furthermore, some family caregivers reported feeling like project managers, regularly assisting with scheduling, and attending medical appointments causing feelings of being overwhelmed with caregiver responsibilities. These important patient- and family-centered considerations were incorporated in design improvements of the appointments section.

#### My Docs

The My Docs (documents) section provides an opportunity to store, review, and share test results, letters, and referrals. Documenting health records [23,25] is important in managing one’s health, and the health service’s HF discharge checklist contains echocardiogram results (Appendix F [21]) for effective communication when moving between care settings. During workshops, the co-design team determined that it was relevant to the patient persona who want to know echocardiogram and pathology results but may periodically misplace this documentation. From the perspective of clinicians, a design priority was to include or track patient data for the purposes of reviewing this data later. The My Docs section was seen as a way for the patient to facilitate communication between the health care team, to better maximize and join care between health care providers and settings.

#### My Team

My Team lists the contact details of the user’s emergency contact person and the health professionals relevant to their care. The recommendations [7] and standards [21] for multidisciplinary care demonstrate the importance of patients engaging effectively with their care providers through, for example, communication with health professionals [24] and documenting their contact details [23-25]. The hospital’s discharge checklist contains specific details regarding the person(s) responsible for postdischarge care and follow-up in the community (Appendix F [21]). This section was considered relevant by all stakeholders throughout the app’s design. Patients may have an available and approachable multidisciplinary team, foster relationships with respect and trust with doctors and nurses in their health care team and seek care regularly. However, participants also reported poor information sharing between health care providers and may be unsure who else is providing care for them commonly relying on memory. The stakeholder map identified that the patient’s spouse and general practitioner are the most likely personal and professional involved in HF self-management. Other members of the family and the pharmacist were also frequently involved, followed by a person’s employer or friends and specialist. In terms of the relevance of the My Team section to health care providers, clinicians wanted a tailored care plan that includes the multidisciplinary care team to ensure that care was holistic, and the design brief emphasizes the importance of managing all stakeholders in care well. Clinicians communicated their concerns of health inequality as some patients have poor access to specific multidisciplinary team members. Finally, during design workshops, the co-design team prioritized team communication as a priority function. These factors resulted in a group decision to include a list of names and details of all persons involved in the care of a person with HF.

### Section 3: My Plan

This section includes nine subsections of HF self-management, and the favorites option and is summarized in Table 3. Each of the nine self-management subsections (listed in no particular order) were included because they are considered as the key in the ongoing management in HF and a relevant, useful, and helpful inclusion by patients, family caregivers, and clinicians. This is based on the local clinical service framework which supports that all patients with HF *should have access to individually tailored, disease management*, and rehabilitation services offered on an outpatient or community basis (p. 29 [21]).

**Table 3:** The My Plan section design and rationale.

Subsection	Item	Rationale
Symptoms	Infographic of common signs and symptoms; help seeking information; understanding deterioration information	Patients reported frequent, varied symptoms. Some were frustrated by multiple, interacting, and complex symptoms or lacked understanding of the treatment rationale in lessening symptom burden; the design brief highlighted the importance of addressing symptom management challenges; the co-design team wanted information and self-help which is visual and simple; source of the infographic was the St Vincent’s Heart Health website [22]; when deciding on the content for the information and advice pages, HF^a^ patient information booklets [23,24], the St Vincent’s Heart Health website [22] and the chronic HF action plan [24] were referred to.
Medications	Medication, previous medications, and allergy list; medicine information; diuretic plan	Clinicians believed medication management should be better supported; patients reported challenges with managing their medications with caregivers often involved; medication information was an important design feature, with specific insights and expertise provided by the pharmacist; the team referred to HF medicine information in patient education booklets [23-25] which includes a medication list template [23], reviewed information on the National Prescriber Scheme Medicine Wise website [26] and the flexible diuretic regime in the hospital’s HF discharge checklist [21].
Fluid	Visual representation of jug at volume of fluid restriction; user enters oral fluid intake throughout the day	Patients experienced challenges with maintaining fluid restrictions; the co-design team wanted tracking with feedback and an interactive interface; fluid-related HF information and advice [24,25,27], local guidelines [22,27], tools and guidelines for documenting fluid intake [23,25,27] and previous qualitative research on fluid restriction adherence [28] were referred to when deciding on content.
Diet	Healthy eating; low salt (sodium) eating including label reading and foods to avoid	Patients wanted general information only; caregivers often prepare meals; specific insights and expertise were provided from the clinical dietitian on the co-design team; information and advice on healthy eating including reducing salt [23-25,29], the *healthy eating* section of the Heart Foundation website [30] were referred to during the design.
Weight	Record daily weight with 7-day graph; interactive, color-coded feedback and pop up alerts	Patients may not be accurate or remember their daily weight; clinicians wanted to include or track HF-related data in an interactive, visual, and tailored format; the cardiac nurse consultant mainly designed the feedback system; information on fluid retention including documenting daily weight and guidelines for help seeking were referred to [23-25,27].
Blood pressure (BP) and Pulse	Record and store BP and pulse measurements	A patient suggested this subsection and the cardiologist supported its inclusion; patient booklets supported intermittent documentation of BP [23,25] and a recent BP and pulse is included in the hospital’s HF discharge checklist [21].
My Future	Information and prompts to *decide* on a plan, *discuss* this with others, speak to your *doctor,* and plan what happens to your *defibrillator*	Clinicians suggested the inclusion of information on advance care planning; the team referred to the local advance care planning website [31] and palliative care recommendations [7].
Well-being	Interactive depression screening tool; *at risk* or *low risk* results screen	This subsection was suggested by a patient; patients frequently reported anxiety and worry; emotional support was a priority function; the team reviewed the local depression screen tool (Patient Health Questionnaire-2, PHQ-2 score) in use at the hospital [32,33] and reviewed psychological care recommendations for HF [7].
Exercise	Step counter with 7-day graph; 3× exercise videos demonstrated by physiotherapist (balance, upper limb, and lower limb) with 7-day graph	Patients reported using their smartphone’s step counter, appreciated supervised physical exercise, and set their own exercise goals; clinicians wanted to include or track data; the physiotherapist designed the exercise program, using the Otago exercise program [34] as a guide.

^a^HF: Heart failure.

#### Symptoms

This subsection includes an infographic containing the common signs and symptoms of HF, information to assist in appropriate help seeking, and information about worsening HF. This subsection was an important inclusion in the app because patients frequently reported symptoms such as breathlessness, urinary frequency, sleep disturbance, fatigue, exhaustion and nighttime breathlessness, anxiety, and agitation. Patients said they were frustrated by multiple, interacting, and complex symptoms. Other patients lacked understanding of the treatment rationale in lessening symptom burden. During workshop activities, the co-design team decided it was a design priority to include information and self-help in a visual and simple format. The infographic representing common HF symptoms was sourced, with approval, from the St Vincent’s Heart Health website [22]. Hyperlinks to further information and advice pages are accessed through this infographic, and it was collated from patient information booklets [23,24], the St Vincent’s Heart Health website [22], the chronic HF action plan [24] as well as the multidisciplinary team members themselves based on their clinical experience and expertise.

#### Medication

The medication component includes a list of current medications, previous medications, an allergy list, medicine information, and the patient’s own diuretic plan. Clinicians thought it appropriate to facilitate improved medication management as a component that should be improved in current care. The pharmacist on the co-design team provided specific insight into the design of this subsection. Patients reported the disruption to their routine when medication prescriptions were changed and the inconsistent documenting of medication lists with some writing changes on scrap paper or even forgetting important changes in the reality of daily life. Family caregivers are sometimes involved in reminders, and patients reported taking tablets with them during outings; so, these realities of the daily management of medications were incorporated into the design of this subsection. During workshop activities where the subsection was further refined, the co-design team members prioritized medication information as an important design feature. The cardiac nurse consultant regularly caring for indigenous Australians saw value in including the color of the medication as a visual reminder. In regard to the literature, medication is a reported important component of HF self-management as per the information contained within the patient education booklets [23-25] which provides a written medication list template [23] and by the hospital literature with the flexible diuretic regime listed in the hospital’s HF discharge checklist (Appendix F [21]). The National Prescriber Scheme Medicine Wise website [26] was also reviewed for general medicine information.

#### Fluid

This subsection comprises of the important fluid restriction guideline for HF. The page displays a visual representation of a measuring jug at the volume of fluid restriction tailored to the patient’s restriction volume in the settings (commonly 1200 mL or 1500 mL per day). The jug gradually fills as users enter oral fluid intake throughout the day. Restricting fluid intake is likely the most important method to prevent fluid congestion alongside taking diuretic medications; however, patients commonly reported challenges with maintaining fluid restrictions in daily life, especially with thirst. Clinicians wanted to include or track data, and during design workshops, the co-design team emphasized that user feedback and an interactive interface were important. Information and advice [24,25,27], local guidelines [22,27], and tools and guidelines regarding documenting fluid intake [23,25,27] were local and national literature sources considered during the design. Previous qualitative research conducted in the same clinical setting regarding fluid restriction adherence was also referred to [28].

#### Diet

The diet component includes information for healthy eating, low salt (sodium) eating, label reading, and foods to avoid. Patients reported that they were not necessarily interested in calorie counting, so general information and advice on healthy eating including reducing salt [23-25,29], recipe suggestions and the *healthy eating* section of Heart Foundation website [30] were consulted. These resources were deemed relevant to family caregivers who commonly prepare and/or assist with meal planning and cooking. Recommendations, advice, and insight regarding nutritional education were provided by the clinical dietitian on the co-design team.

#### Weight

Daily weight management in this subsection of the app gives the user the ability to record daily weight, view a 7-day weight trend on a bar graph, receive color-coded feedback based on this data and pop up alerts depending on stability of that day’s weight in comparison to the dry weight set in the settings tab. Information on fluid retention including documenting daily weight and guidelines for help seeking [23-25,27] throughout the literature was consulted, alongside specialist input from the 2 nurse practitioners on the co-design team who regularly assisted in managing the variations in weight due to fluid congestion in worsening HF. The patients interviewed had variable understandings regarding fluid management, reporting what they knew about dry weight and the concern about going 2 kg over their dry weight. Clinicians thought that the weight section was highly important to include in the apps design, specifically around tracking weight data over the longer term. The co-design team believed having an interactive and visual interface that was tailored to patient parameters improved its utility. The nurse consultant was particularly involved in the colorful design of the feedback alert system when weights varied from the dry weight.

#### Blood Pressure and Pulse

For some, self-monitoring of BP and pulse is important in HF. This *My Plan* subsection provides the option to record and store BP and pulse measurements. Clinicians generally supported the inclusion of patient data to track them; however, the inclusion of recording BP and pulse specifically, was inconsistent. One patient initially suggested the inclusion and the cardiologist agreed to it; however, the other clinicians believed it was not important enough to include especially comparative to other daily measures to track in HF. A second patient who does not self-monitor this data did not see it necessary to include in the app. Through ongoing discussions, it was decided this subsection would be included in the final design as the literature supports the intermittent documentation of BP [23,25]. Furthermore, the hospital’s discharge checklist specifies recording a postural BP (measurements taken while sitting and standing) and nature of the pulse as either regular, irregular, or paced (Appendix F [21]).

#### My Future

This subsection relates to the long-term planning required for patients with HF. This section contains information and prompts to decide on a plan, discuss this with others, speak to their doctor, and plan what happens to their defibrillator (an implantable medical device) if they have one. The inclusion of this section was deemed relevant by clinicians, and patients on the co-design team agreed to its inclusion without providing specific input into its content. The team reviewed the local advance care planning website [31] and palliative care recommendations for the multidisciplinary care of people with HF [7] as key literature sources.

#### Well-Being

The well-being component represents the psychological aspect of self-management. It contains an interactive depression screening tool, *at risk* or *low risk* results screens and information and advice pages. This section was suggested by a patient on the co-design team during the second design workshop as they felt it necessary to address the emotional support needed for people living with HF. In the interviews, patients reported the frequency of anxiety and worry. Patient needs included family, nature, mindfulness, and happiness demonstrated in 1 patient persona who balances quality of life with safety in HF in her pursuit to maintain well-being. The co-design team then conducted a literature review of the psychological care recommendations for HF [7], and clinicians communicated their use of the depression screen tool (Patient Health Questionnaire-2, PHQ-2 score) [32,33] routinely used in current practice. The PHQ-2 store is a 2-item validated questionnaire designed for the initial assessment of depression and anxiety in the primary care setting [32,33] and precedes referral for specialized care in the local hospital setting.

#### Exercise

The exercise component has a step counter with 7-day graphical representation of daily steps. There was an inclusion of 3 exercise videos demonstrated by the physiotherapist—balance, upper limb, and lower limb—with a 7-day graph. Patient interviews uncovered that patients were using their smartphone’s step counter, appreciate supervised physical exercise and set exercise goals, for example one patient setting a 2 km daily walk. Clinicians valued ability to track patient data in considering the relevance to the patient group, and physiotherapist on the co-design team designed the exercise program to the specific context. The Otago exercise program to prevent falls in older adults [34], a resource commonly referred to for this patient population, formed the basis of the content of the balance and lower-limb exercises.

## Discussion

### Principal Findings

We have presented the final design of the *Care4myHeart* app which includes the home screen, a health management section, and a My Plan section. With the goal to support local patients with HF self-management and representing the opinions and perspectives of those who would use or recommend the novel app, we enlisted a co-design methodology. The strength of the context-specific co-design process to elicit the final design was the access to, and ongoing involvement of, key stakeholders and the relevant literature. However, the strength of context specificity is also a limitation to portability, and the final design is limited to the stakeholders involved in its development. These key strengths and limitations are explained further.

### Strengths of the Co-Design Process to Achieve the Final Design

The greatest strength of the co-design process to achieve the design outcomes was the involvement of clinicians, patients, and family caregivers. Drawing on best practice, the literature supports using collaborative, team-based processes to develop mHealth interventions [35]. The benefit of the approach to design was strategically coordinating stakeholder involvement within each development stage. As we progressed from the empathize and define phase to the ideate and prototype phase, we were able to achieve the intermediate design goals to input into the subsequent phases, ensuring efficiency of development to achieve the final design.

First, in the empathize and define phase, stakeholders were individually interviewed to understand their experiences, ensuring perspectives and opinions were appropriately defined. Appreciating the various interests of different stakeholders [35] by interviewing patients, caregivers and clinicians separately ensured a good understanding of health care challenge to be addressed in the design from many different standpoints. However, it was the careful emphasis on the *define* phase—where these experiences were visually represented in poster format—which facilitated cross-stakeholder empathy. Referred to as a mutual learning [36], knowledge transfer between different stakeholders was maximized [35] in this process. Patient personas were a way to represent the important health care consumer voice, as patients are often passive in health care improvement activities [37] and traditionally excluded from design efforts [17]. It has previously been shown that the benefit of documenting patient narratives on preferences, beliefs, and values is that it legitimizes their preferences [38]. Equally, it was important to interview caregivers in HF, who in other settings have expressed distrust towards the health system due to feelings of role strain [39]. As a vessel for positive change in health care, the empathize and define phase in co-design presents a method of inclusion and mutual respect, ensuring that for caregivers (and indeed all stakeholders) are *more explicitly involved in the design of disease-management interventions* as recommended by Burke and colleagues (p. 736 [39]). The benefit of representing stakeholder experiences separately gives relevance to their specific needs and insights to be considered in the subsequent design stage.

Second, bringing stakeholders together was beneficial in the ideation phase for a fit-for-purpose design. Collaborative practices support design features that would be accepted by potential users and are technically feasible [35]. As suggested by Skeels and Pratt [36], the role of team members as *partners* in the design process was emphasized in our design process, allowing for the creation of a collaborative group dynamic where participants addressed each other directly [36] in design workshops. However, in this research, we were limited by the small number of patients who chose to attend the workshops. To account for this, design activities included the use of the design artefacts, commonly used in design workshops as a design strategy to provoke an alternate way of thinking, challenge perceptions or raise questions about conventions and assumptions [17]. Design artefacts were considered a practical tool for co-design, spurring creativity, and supporting meaningful participation [16] through discussion and collaborative decision making to achieve the conceptual design of the app by the end of the second workshop.

Finally, in the prototype phase, all stakeholders provided feedback independently to refine the wireframes. The overarching principal was that the design reflected the ideas generated by the group [36] even though stakeholder involvement was done individually. Content was written by clinicians with the relevant expertise, checked by patients for clarity, and iteratively refined until consensus was achieved. One-to-one feedback sessions facilitated a hands-on assessment of the digital prototype version for review. To maximize honest feedback and in appreciation of their voluntary participation, the nurse-lead offered a safe, respectful, and relaxed environment. Updating the prototype quickly meant they were engaged and valued in the creation of the innovation.

Another noteworthy contribution of this research was incorporating the literature, guidelines, and current practices into the design which strengthened the relevance of the app to the health care context. Clinicians aspired to develop this app as a self-management tool to be an adjunct offering in addition to existing HF care. To support clinicians in providing the expert care they aspired to provide, they were unanimous, it needed to include the locally relevant evidence-based information and be consistent with the self-management support literature they provide. The app aimed to supplement (not replace) other traditional formats of patient education (eg, patient information booklets [23-25]) as interventions that emphasize and reinforce the complexity of HF have been considered particularly valuable [4]. Anderson and Emmerton [5] suggest pairing app interventions with health care professional input, advising against *leaving consumers to their mobile devices without periodic check-ups* (p. 594 [5]). The purposeful integration of the app to the health care setting is undoubtedly more likely to be achieved if it is developed within an existing health care environment, with only 2% of existing consumer mHealth apps connecting and communicating with provider health systems [10]. Embedded practices and policies were, therefore, incorporated from early in the app’s design to ensure consistency with the local execution of evidence-based care.

### Limitations of the Co-Design Process

The outcomes of the design are limited by the stakeholders involved in the project. Each person had a role to play to positively impact the final design but also the potential to limit the design. For example, the nurse lead who facilitated the design activities had limited design experience and, thus, learned co-design processes as the project developed. A skilled facilitator in co-design chooses the right tools and provides the right environment to engage and inspire [16]. In addition, study participants were drawn from local clinicians, patients, and family caregivers who were a self-selecting group of volunteers. Therefore, the design outcomes are based perspectives from this limited, context-specific group of stakeholders, which would have biased the findings. From an organizational point of view, the hospital or university venture needed to be formalized as a research project which had implications on recruitment. In this case, ethical approval was required to obtain patient and family caregiver participation which means that not all target end users could be involved. Recruited participants were those with adequate literacy to understand the information sheet and consent form, and confidence to collaboratively engage with various stakeholders, many of whom are in positions of power in the health care setting. Future co-design projects should incorporate more diverse patient and family caregiver perspectives to ensure the health technology is relevant to as many consumers as possible and not limited in relevance to a homogenous patient population.

The strength of context specificity is also a limitation of the portability of the design. The Australian policy and current practices and procedures were included to address the needs of the local health care environment. Therefore, extra work in the design will be required to make the app relevant outside of the community in which it was designed, to be aligned with other health care environments and consumer needs.

### Future Directions

The first step is assessing patients’ acceptance of such a tool to their current lifestyle. This research team has undertaken a usability study aimed to understand the experience of using the app with new subset of patients not involved in the design phase. Findings from the usability study will determine other features for inclusion in the next version and provide implications of consumer mHealth apps to self-management practices.

Co-design processes for context-specific digital health, particularly with the involvement of multiple stakeholders, should be evaluated for effectiveness. Currently, researchers are interrogating the process from the perspective of co-design participants and the nurse lead.

### Conclusions

In this paper, the final, modular design of the consumer mHealth app for HF, *Care4myHeart*, was presented with the rationale associated with each app section and subsection. The design outcomes were elicited from a co-design process incorporating the active involvement of patients, family caregivers, and clinicians together with the local literature. In planning for utility and acceptability, health app development teams should strategically incorporate relevant stakeholders and the literature to design mHealth solutions which are rigorously designed from a solid evidence base and relevant to those who will use and recommend their use.

## Abbreviations

BP: blood pressure
HF: heart failure
mHealth: mobile health
PHQ-2: Patient Health Questionnaire-2
